# Implementation of pharmacogenetic testing in oncology: *DPYD*-guided dosing to prevent fluoropyrimidine toxicity in British Columbia

**DOI:** 10.3389/fphar.2023.1257745

**Published:** 2023-09-08

**Authors:** Angela Wu, Helen Anderson, Curtis Hughesman, Sean Young, Caroline Lohrisch, Colin J. D. Ross, Bruce C. Carleton

**Affiliations:** ^1^ Department of Experimental Medicine, University of British Columbia, Vancouver, BC, Canada; ^2^ BC Children’s Hospital Research Institute, Vancouver, BC, Canada; ^3^ Medical Oncology, BC Cancer, Provincial Health Services Authority, Vancouver, BC, Canada; ^4^ Cancer Genetics and Genomics Laboratory, BC Cancer, Provincial Health Services Authority, Vancouver, BC, Canada; ^5^ Department of Pathology and Laboratory Medicine, University of British Columbia, Vancouver, BC, Canada; ^6^ Faculty of Pharmaceutical Sciences, University of British Columbia, Vancouver, BC, Canada; ^7^ Division of Translational Therapeutics, Department of Pediatrics, University of British Columbia, Vancouver, BC, Canada; ^8^ Department of Medical Genetics, University of British Columbia, Vancouver, BC, Canada; ^9^ Therapeutic Evaluation Unit, Provincial Health Services Authority, Vancouver, BC, Canada

**Keywords:** adverse drug reactions, clinical implementation, DPYD, fluoropyrimidine toxicity, pharmacogenetics

## Abstract

**Background:** Fluoropyrimidine toxicity is often due to variations in the gene (*DPYD*) encoding dihydropyrimidine dehydrogenase (DPD). *DPYD* genotyping can be used to adjust doses to reduce the likelihood of fluoropyrimidine toxicity while maintaining therapeutically effective drug levels.

**Methods:** A multiplex QPCR assay was locally developed to allow genotyping for six *DPYD* variants. The test was offered prospectively for all patients starting on fluoropyrimidines at the BC Cancer Centre in Vancouver and then across B.C., Canada as well as retrospectively for patients suspected to have had an adverse reaction to therapy. Dose adjustments were made for variant carriers. The incidence of toxicity in the first three cycles was compared between *DPYD* variant allele carriers and non-variant carriers. Subsequent to an initial implementation phase, this test was made available province-wide.

**Results:** In 9 months, 186 patients were tested and 14 were found to be heterozygous variant carriers. Fluoropyrimidine-related toxicity was higher in *DPYD* variant carriers. Of 127 non-variant carriers who have completed chemotherapy, 18 (14%) experienced severe (grade ≥3, Common Terminology Criteria for Adverse Events version 5.0). Of note, 22% (3 patients) of the variant carriers experienced severe toxicity even after *DPYD*-guided dose reductions. For one of these carriers who experienced severe thrombocytopenia within the first week, *DPYD* testing likely prevented lethal toxicity. In *DPYD* variant carriers who tolerate reduced doses, a later 25% increase led to chemotherapy discontinuation. As a result, a recommendation was made to clinicians based on available literature and expert opinion specifying that variant carriers who tolerated two cycles without toxicity can have a dose escalation of only 10%.

**Conclusion:** *DPYD*-guided dose reductions were a feasible and acceptable method of preventing severe toxicity in *DPYD* variant carriers. Even with dose reductions, there were variant carriers who still experienced severe fluoropyrimidine toxicity, highlighting the importance of adhering to guideline-recommended dose reductions. Following the completion of the pilot phase of this study, *DPYD* genotyping was made available province-wide in British Columbia.

## 1 Introduction

It is estimated that two million cancer patients worldwide are treated with fluoropyrimidines every year. This group of drugs includes 5-fluorouracil (5-FU) and capecitabine, the latter being a prodrug metabolized into 5-FU (Longley et al., 2003; Walko and Lindley, 2005). Fluoropyrimidines are a cornerstone of chemotherapy, commonly prescribed for adjuvant and palliative treatment of multiple solid tumour types, including colorectal, head and neck, gastric, pancreatic, and breast cancers. However, 10%–40% of the treated population develop severe treatment-related toxicity, leading to hospitalization, treatment discontinuation, or even death in approximately 1% of patients (Hoff et al., 2001; Twelves et al., 2005; André et al., 2015; Amstutz et al., 2018). 10% of patients develop these severe toxicities within the first three chemotherapy cycles and are obliged to discontinue therapy (Meulendijks et al., 2016b). The most common fluoropyrimidine-induced adverse events are diarrhea, nausea, vomiting, mucositis, myelosuppression, and hand-foot syndrome (HFS) (Negarandeh et al., 2020).

One of the best-studied and recognized causes of fluoropyrimidine-related toxicity is the reduced activity of the primary 5-FU inactivating enzyme dihydropyrimidine dehydrogenase (DPD), caused by genetic variants in *DPYD*, the gene encoding DPD. The interindividual variability in fluoropyrimidine metabolism by DPD results in toxic concentrations of drug exposure in some patients and not others. It has been repeatedly demonstrated in retrospective and prospective studies that patients with DPD deficiency receiving standard doses of fluoropyrimidine therapy are at a significantly higher risk of severe toxicity (van Kuilenburg, 2004; Terrazzino et al., 2013; Rosmarin et al., 2014; Meulendijks et al., 2015; Toffoli et al., 2015; Barin-Le Guellec et al., 2020; Deng et al., 2020). The most well-studied polymorphism is DPYD*2A (c.1905 + 1G>A), which results in a complete loss of function. Approximately 1.6% of individuals of European ancestry are heterozygous carriers of DPYD*2A and thus have a 50% reduction in DPD function relative to those with two completely functional alleles (Deenen et al., 2016; Amstutz et al., 2018). Additionally, there are three other *DPYD* variants with a statistically significant association with severe 5-FU toxicity that are deemed clinically relevant: the loss of function allele DPYD*13 (c.1679T>G) as well as the reduced function alleles c.2846A>T and c.1236G>A (HapB3) (Amstutz et al., 2018; Henricks et al., 2018). Together, these four alleles may be found in 3%–8% of those of European ancestry. Expert groups have recommended testing for these four variants (Amstutz et al., 2018; Hamzic et al., 2020a; Lunenburg et al., 2020).

Many other *DPYD* variants that impact DPD phenotype *in vitro* are extremely rare and have not been identified in large cohort studies (Offer et al., 2014; Amstutz et al., 2018). Two additional variants were considered due to the ancestrally diverse Vancouver population. Fluoropyrimidine toxicity may be seen in patients carrying c.557A>G, a reduced function variant present in 3% of individuals with African ancestry, as well as c.2279C>T, a reduced function variant present in 1% of individuals with South Asian ancestry (Offer et al., 2013; 2014; Elraiyah et al., 2017).

In a prospective trial that screened for DPYD*2A in 2038 patients, Deenen et al. demonstrated that a 50% dose reduction for variant carriers reduced the frequency of severe fluoropyrimidine-related toxicity from 73% to 28%, which was comparable to the 23% rate of toxicity observed in non-carrier patients treated with a full dose (Deenen et al., 2016). In another large prospective trial, Henricks et al. screened for all four aforementioned “European” *DPYD* variants. They found that an upfront dose reduction of 50% in heterozygous c.1905 + 1G>A carriers reduced the frequency of severe toxicity from 77% to 18% (Henricks et al., 2018). Importantly, Henricks et al. also found that a 25% dose reduction in c.1236G>A and c.2846A>T carriers was insufficient, as the risk of severe toxicity (39% in c.1236G>A and 47% in c.2846A>T) remained elevated relative to non-carriers of the *DPYD* variants. Consequently, the fluoropyrimidine dosing guidelines by the Clinical Pharmacogenetics Implementation Consortium (CPIC) now recommends a 50% initial dose reduction for patients who are heterozygous for either reduced function or non-functional *DPYD* variants (Amstutz et al., 2018).

Importantly, these trials demonstrated that dose reductions of capecitabine and 5-FU in *DPYD* variant carriers likely did not underdose patients. This has been demonstrated by pharmacokinetic analyses showing similar levels of drug exposure in heterozygous carriers treated with reduced doses and non-carriers treated with standard doses (Henricks et al., 2018). Moreover, retrospective studies have shown that *DPYD* variant carriers experience up to an 88% increased risk of grade ≥ 3 fluoropyrimidine toxicity (van Kuilenburg et al., 2000; Van Kuilenburg et al., 2002). Given a relatively high carrier frequency, a large group of at-risk patients can be pre-emptively identified through prospective genotyping (Amstutz et al., 2018). Prospective data show lower toxicity with properly informed genotype-guided dosing with no consequent decrease in survival (Henricks et al., 2019). Furthermore, it has been shown that genotype-guided dosing results in equivalent systemic fluorouracil concentrations across all dosing schedules (Deenen et al., 2016; Henricks et al., 2018; 2019).

Currently, routine prospective *DPYD* genotyping is not the standard of care in North America, although recent implementation trials have emerged (Reizine et al., 2020; Varughese et al., 2022; Baker et al., 2023). The National Comprehensive Cancer Network (NCCN), the American Society of Clinical Oncology (ASCO), and the US Food and Drug Administration (FDA) do not explicitly support routine pre-treatment *DPYD* testing. However, it is important to also note that not every agency agrees that the lack of pre-emptive testing guidance from NCCN or the labeling by the FDA is appropriate. The European Medicines Agency, the French National Agency for the Safety of Medicines and Health Products, and the Medicines and Healthcare Products Regulatory Agency (United Kingdom) have each approved guidelines for pre-emptive determination of DPD activity for patients treated with fluoropyrimidines (Medicamentet des Produits de Sante, 2019; European Medicines Agency, 2020; NHS England, 2020; UK Chemotherapy Board, 2020). Expert commentaries recently published discuss the lack of support for *DPYD* testing before fluoropyrimidine chemotherapy in North America (Hertz, 2022; Baker et al., 2023; Hertz et al., 2023). They cite the FDA labelling which acknowledges that patients with DPD deficiency have an increased risk of life-threatening toxicity, but instead of recommending testing, FDA suggests an unlikely scenario in which patients who have known DPD deficiency should discuss it with their physicians. The authors are oncologists, clinical pharmacists, and other experts affiliated with various oncology centres and conclude with a recommendation for standard of care pre-treatment *DPYD* testing and urging NCCN, ASCO, and FDA to update their guidelines. It is also notable that the Dana Farber Cancer Institute (Boston, United States) launched a program to promote *DPYD* testing in 2022; Oregon Health and Science University paid USD$1M to settle a lawsuit from a widow of a patient after DPD deficiency was implicated in the fatality.

In Canada, other methods to determine DPD function such as therapeutic drug monitoring (TDM) and phenotyping have not been implemented to our knowledge. In the absence of prospective screening, physicians are blind to the necessity of dose reduction, inevitably resulting in an avoidable increase in the incidence of severe toxicity. Prior to this initiative, prospective *DPYD* genotyping was not routinely available in British Columbia (BC). As such, patients predisposed to fluoropyrimidine toxicity could not be identified prior to initiation of therapy, resulting in a preventable increase in morbidity and mortality.

## 2 Methods

A testing program was developed for genotype-guided dosing of fluoropyrimidines based on six variants (Table 1). This program was successfully implemented at BC Cancer Vancouver Centre in August 2022 and served as a model for the program’s provincial expansion in May 2023. The program’s central aim was to prevent severe fluoropyrimidine-related adverse drug reactions through *DPYD* genotype-guided dose individualization. An additional aim was to provide prospective data upon which to refine genotype-based dosing strategies, including collecting prospective data for *DPYD* variants prevalent in those of non-European ancestries (c.557A>G, c.2279C>T).

**TABLE 1:** *DPYD* allele panel for testing with activity score and prevalence. Allele function, activity score, and variant frequencies obtained from Amstutz et al., 2018.

*DPYD* variant	Allele function	Activity score (Amstutz et al., 2018)	Variant frequency in different ancestries					
			European	Afro-caribbean	Sub-saharan African	East Asian	South Asian	Latino
c.1905 + 1G>A (DPYD*2A)	Non-functional	0	0.008	0.003	0.000	0.000	0.005	0.001
c.1679T>G (DPYD*13)	Non-functional	0	0.001	0.000	0.000	0.000	0.000	0.000
c.2846A>T	Reduced function	0.5	0.004	0.003	0.000	0.000	0.001	0.002
c.1236G>A (HapB3)	Reduced function	0.5	0.024	0.003	0.000	0.000	0.020	0.006
c.557A>G	Reduced function	0.5	0.000	0.012	0.026	0.000	0.000	0.001
c.2279C>T	Reduced function	0.5	0.000	0.000	0.000	0.000	0.006	0.000

### 2.1 Study design and participants

The purpose of this study was to integrate *DPYD* testing into the clinical workflow and collect prospective data during the first 9 months of implementation. This was an observational clinical trial performed at the BC Cancer Vancouver Centre. All adult patients (18 years or older) scheduled to start on a fluoropyrimidine-based chemotherapy regimen were eligible for enrolment (expected n = 20–30/month) through their treating oncologist.

Research ethics board approval was obtained in June 2022. Prospective *DPYD* genotyping became available to BC Cancer Vancouver patients at the end of August 2022. All patients provided written, informed consent prior to study enrolment. Informed consent was also obtained for biobanking samples to allow for future exome or whole genome sequencing to identify novel rare variants. *DPYD* testing for all patients in this study was covered by the provincial healthcare system.

### 2.2 Procedures

**FIGURE 1:**
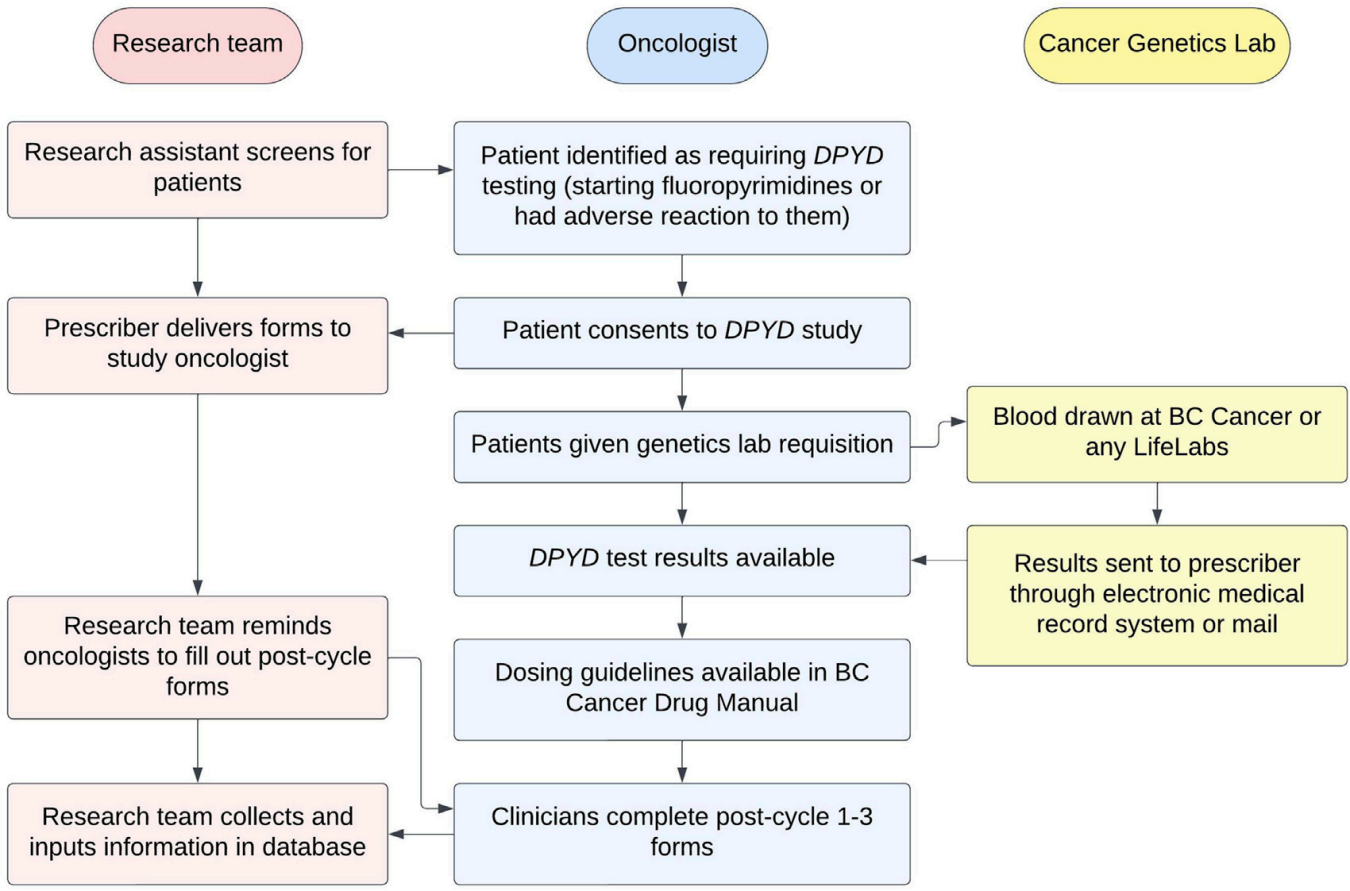
Implementation workflow for *DPYD* genotyping in British Columbia.

An implementation workflow was developed that outlined the study processes and roles of the research team, medical oncologists, and the Cancer Genetics and Genomics Laboratory (Figure 1).

Patients were consented prior to the start of chemotherapy and genotyped for six *DPYD* variants DPYD*2A, DPYD*13, c.2846A>T, c.1236G>A, c.557A>G, c.2279C>T (Table 1). If they consented, patients reported their ancestry as their parents’ and grandparents’ countries of origin. If they did not know of specific countries, patients gave a broad categorization of their ancestry (e.g., of European origin). Patients then received a paper requisition signed by their medical oncologist and had their blood drawn centrally (BC Cancer) or at any community procurement centre in British Columbia. Pursuant to routine clinical practice, specimens were forwarded to the Cancer Genetics and Genomics Laboratory for testing and subsequent clinical reporting.

Genomic DNA (gDNA) was extracted from 150 µL of isolated buffy coat using the Maxwell RSC Buffy Coat DNA Kit and Maxwell RSC instrument (Promega, Wisconsin, United States). *DPYD* testing was performed by quantitative PCR (qPCR) on a QuantStudio 7 Pro real-time PCR system (ThermoFisher, Massachusetts, United States) using multiplexed genotyping reactions containing 10 ng of Nanodrop (ThermoFisher) quantified gDNA loaded into each well. Custom primers and probes (sequences available upon request) were designed to allow for the six *DPYD* variants to be genotyped in 3 multiplexed reactions. *DPYD* Multiplex Set gDNA (SensID, Rostock, Germany) as well as laboratory-developed controls, prepared from gBlocks (IDT, Iowa, United States) and cell lines along with a no template control were included in each run. For any given variant, the controls were prepared such that they were representative of a homozygous wild-type, heterozygous, or homozygous result and used to classify the *DPYD* genotypes of patient samples using the Genotyping module on the QuantStudio Real-time PCR Software v1.7.2 (ThermoFisher). With a median turnaround time of 6 days, the report was uploaded into the patient’s electronic medical record.

The results report included a patient’s *DPYD* genotype as well as a predicted activity score (see Supplementary Material for examples of reports). The activity score was clearly noted to be a prediction as only six *DPYD* variants were tested. For patients carrying rarer, untested, variants that impair DPD function and were not tested, this estimate would not be a true reflection of DPD activity. A dosing table based on the CPIC guideline recommendations is available online, both in the Cancer Drug Manual^©^ Appendix and Drug Index for capecitabine and 5-FU (Table 2). Per the CPIC guidelines, a reduced function *DPYD* allele has an activity score of 0.5, and a non-functional allele has a score of 0, with the total activity score being additive (Amstutz et al., 2018). According to the table, intermediate metabolizers with activity score 1.0 were given upfront 50% dose reductions. In practice, given the elevated toxicity and elevated metabolite levels when dose was only reduced by 25% for patients with an activity score of 1.5 (Henricks et al., 2018), oncologists gave 50% dose reductions to patients with an activity score of 1.5 and the simplicity of this guideline also facilitated implementation (Table 2).

**TABLE 2:** Dosing table for capecitabine and 5-fluorouracil based on *DPYD* activity score. Clinical Pharmacogenetics Implementation Consortium (CPIC) guidelines are linked, and the latest guideline recommendation that patients who are homozygous for c.2846A>T may require more than 50% in the starting dose is noted.

Predicted activity score	Genotype	Likely *DPYD* phenotype	Dosing Guidelines for Fluoropyrimidines
0	Homozygous (or compound heterozygous) for a non-functional variant	Poor metabolizer	Do not use
0.5	One non-functional + one reduced function variant		Use not recommended. If alternative agents are not a suitable therapeutic option, administer at a strongly reduced dose (at least 75% reduction) with early therapeutic drug monitoring
1.0	Heterozygous for a non-functional variant	Intermediate metabolizer	A 50% lower starting dose is recommended. Titrate future doses based on clinical judgement
	Homozygous for a reduced function variant*		
1.5	Heterozygous for a reduced function variant		A 25%–50% lower starting dose is recommended. Titrate future doses based on clinical judgement
2.0	Variant negative	Normal metabolizer	No indication for changing dose

De-identified clinical and demographic data were stored within a Research Electronic Data Capture (REDCap) database. Data were collected on cancer diagnosis, comorbidities, standard laboratory assessments as part of routine clinical care, *DPYD* results and chemotherapy details and toxicities experienced. Fluoropyrimidine-induced toxicities recorded included neutropenia, thrombocytopenia, diarrhea, nausea, vomiting, hand-foot syndrome, mucositis and chest pain. Any other toxicities were also recorded. Toxicity data was reported by patients’ treating oncologists for the first three cycles by completing standardized forms listing toxicity grading according to the National Cancer Institute Common Terminology Criteria for Adverse Events v5.0 (CTCAE) (US Department of Health and Human Services, 2017). These forms were completed during post-cycle follow-up visits when oncologists asked patients about each toxicity. Hospitalization and unscheduled medical encounters, general practitioner visits, urgent care visits, and phone calls to the BC Cancer nursing line that were the result of fluoropyrimidine toxicities were also recorded.

### 2.3 Outcomes

The primary endpoint of the study was the proportion of *DPYD* variant carriers experiencing severe (CTCAE grade ≥3) toxicity compared to that of variant-negative patients. Other endpoints were implementation-related outcomes, including the proportion of doses modified in response to genotype-guided dose recommendations and the proportion of *DPYD* test results returned before chemotherapy initiation. The uptake of testing by oncologists was assessed by comparing the number of patients tested and enrolled in the study to the number of new patients starting fluoropyrimidine-based protocols. Exploratory endpoints were the dose intensity of fluoropyrimidines for *DPYD* variant carriers compared to variant-negative patients and the variant distribution and incidence of toxicity by self-reported ancestry. The relative dose intensity was defined as the given dose divided by the standard dose for the patient’s indicated regimen.

### 2.4 Statistics

Baseline characteristics between patients with and without the tested *DPYD* variants were compared through descriptive statistics. Normality was assessed for age (*p* < 0.05 using Shapiro-Wilk), so nonparametric methods were used to compare age between variant and non-variant carriers. The difference between *DPYD* variant and non-variant carriers was determined using Mann-Whitney U tests for age. Fisher’s exact tests were used for dichotomous outcomes. A *p*-value < 0.05 was considered significant.

## 3 Results

Between September 2022 to May 2023, 186 patients received *DPYD* testing (Table 3). The most common tumour types were colorectal (n = 80; 52%), breast (n = 21, 14%), and pancreatic (n = 18, 12%). The most common self-reported ancestries were European (n = 84; 55%) and East Asian (n = 26, 17%). There were 14 (8%) heterozygous *DPYD* variant allele carriers. The cohort had no significant differences in sex, age, tumour type, tumour stage, and treatment regimen between *DPYD* variant and non-variant carriers.

**TABLE 3:** Clinical and demographic characteristics for n = 186 cancer patients who underwent *DPYD* testing for treatment with fluoropyrimidine-based regimens. Significance between *DPYD* variant and non-variant carriers was determined using Mann-Whitney U tests for age, and Fisher’s exact test for sex, ancestry, tumour type, and regimen type. A *p*-value < 0.05 was considered significant.

	Overall (n = 186)	Variant carriers (n = 14)	Non-variant carriers (n = 172)	*p*-value
Sex				
Male	87 (47%)	7 (50%)	82 (48%)	1.00
Age				
Median (IQR)	63 (56–73)	66 (54–74)	63 (56–73)	0.63
**Ancestry** ^a^	—	—	—	0.14
European	113 (61%)	13 (93%)	100 (55%)	—
African	2 (1%)	1 (7%)	1 (1%)	—
East Asian	31 (17%)	0 (0%)	31 (18%)	—
South Asian	14 (8%)	0 (0%)	14 (8%)	—
Indigenous	5 (3%)	0 (0%)	5 (3%)	—
Hispanic	4 (2%)	0 (0%)	4 (2%)	—
Mixed (European, East Asian, African)	6 (3%)	0 (0%)	6 (3%)	—
Declined to report	11 (6%)	0 (0%)	11 (6%)	—
**Tumour type**	—	—	—	0.52
Colorectal	98 (53%)	6 (43%)	92 (53%)	—
Gastric	9 (5%)	0 (0%)	9 (5%)	—
Esophageal	6 (3%)	1 (7%)	5 (3%)	—
Pancreatic	23 (12%)	2 (14%)	21 (12%)	—
Anal	9 (5%)	2 (14%)	7 (4%)	—
Bile duct	13 (7%)	1 (7%)	12 (7%)	—
Breast	22 (12%)	2 (14%)	20 (12%)	—
Other (gallbladder, liver, unknown primary)	6 (3%)	0 (0%)	6 (3%)	—
**Tumour staging**	—	—	—	0.67
I	5 (3%)	0 (0%)	5 (3%)	—
II	27 (15%)	3 (21%)	24 (14%)	—
III	85 (47%)	5 (36%)	80 (47%)	—
IV	69 (37%)	6 (43%)	63 (37%)	—
**Treatment regimen type**	—	—	—	0.88
5-FU combination therapy	59 (32%)	4 (29%)	55 (32%)	—
Capecitabine monotherapy	44 (24%)	3 (21%)	41 (24%)	—
Capecitabine + platinum agent	45 (24%)	3 (21%)	42 (24%)	—
Capecitabine + other anticancer drugs	4 (2%)	0 (0%)	4 (2%)	—
Capecitabine + radiotherapy	34 (18%)	4 (29%)	30 (17%)	—
*DPYD* status				
Non-variant carrier	172 (92%)	0 (0%)	172 (100%)	—
DPYD^a^2 heterozygous	3 (2%)	3 (21%)	0 (0%)	—
DPYD^a^13 heterozygous	1 (1%)	1 (7%)	0 (0%)	—
c.1236G>A heterozygous	7 (4%)	7 (50%)	0 (0%)	—
c.2846A>T heterozygous	2 (1%)	2 (14%)	0 (0%)	—
c.557A>G heterozygous	1 (1%)	1 (7%)	0 (0%)	—
c.2279C>T heterozygous	0 (0%)	0 (0%)	0 (0%)	—

^a^ This study was underpowered to detect ancestral differences.

**FIGURE 2:**
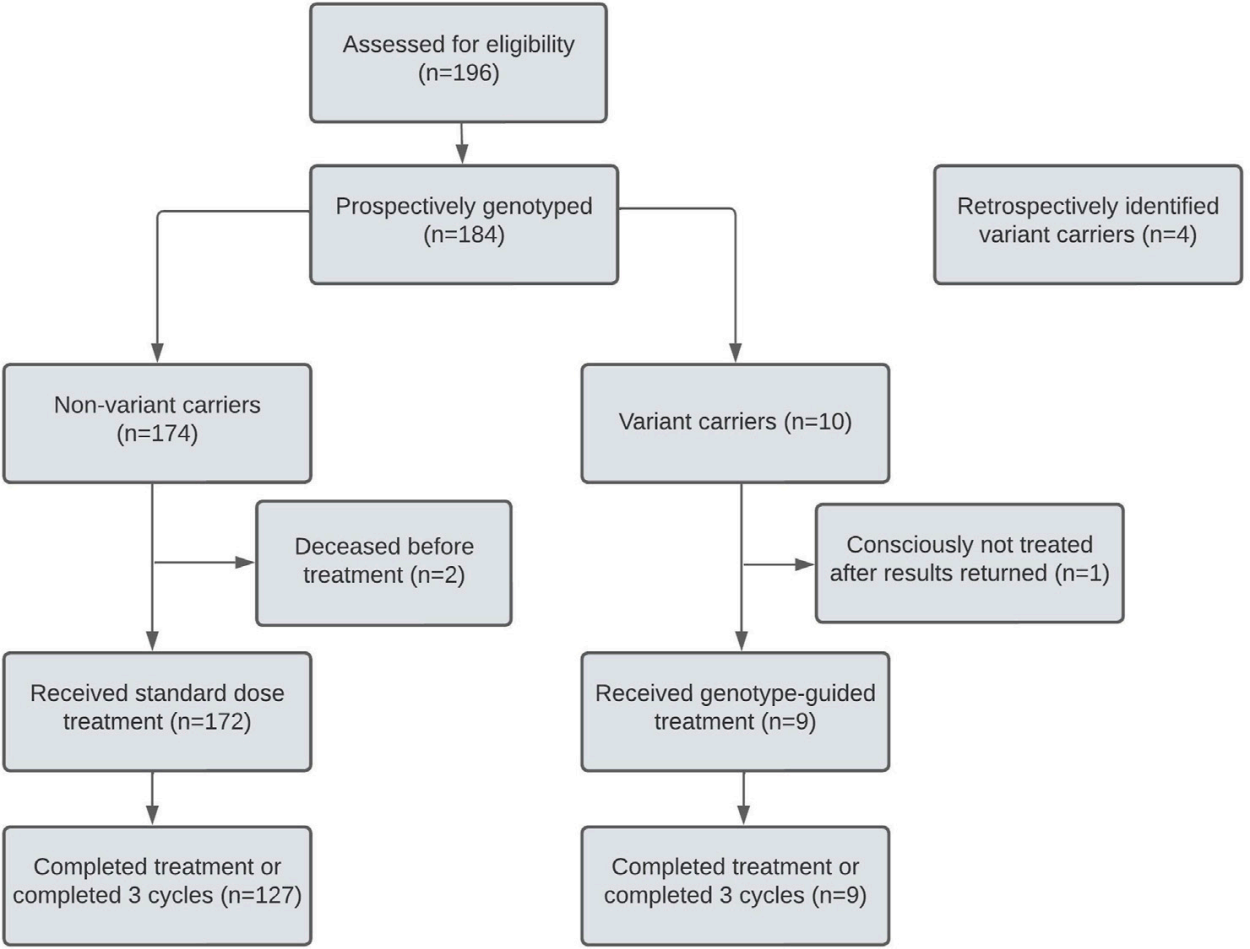
CONSORT diagram.

Out of the 14 *DPYD* variants identified in this cohort, four were retrospectively discovered after discontinuing chemotherapy due to fluoropyrimidine toxicity (Figure 2). Out of the remaining 10 prospectively identified variant carriers, three *DPYD* variant carriers received their results after initiating chemotherapy. One patient tolerated the first cycle at the full dose, but the physician opted to discontinue fluoropyrimidine-based therapy, citing limited benefit and increased toxicity risk with future cycles. The other two patients received guideline-recommended dose reductions in cycle 2 after their results were returned and one experienced severe toxicity (CTCAE grade 3) despite the reduced dose. As these two patients promptly received dose reductions after a cycle, they were included in the analysis as dose-reduced *DPYD* variant carriers (Table 4). Seven *DPYD* variant carriers received their pharmacogenetic reports before the first cycle of chemotherapy and were dose-reduced by 50% for cycle 1.

**TABLE 4:** Toxicity outcomes in patients within the first three cycles of chemotherapy. Significance between *DPYD* variant and non-variant carriers was determined using Fisher’s exact tests. A *p*-value < 0.05 was considered significant.

	Dose-reduced *DPYD* variant carriers (n = 9)	Non-variant carrier (n = 127)	*p*-value
Completed chemotherapy up to 3 cycles			
Overall grade 3	2 (22%)	18 (14%)	0.62
GI toxicity grade 3	0 (0%)	12 (8%)	1.00
HFS grade 3	1 (11%)	0 (0%)	0.066
Hematologic toxicity grade 3	1 (11%)	6 (5%)	0.39
Overall grade 2	3 (33%)	34 (27%)	0.70
GI toxicity grade 2	2 (22%)	23 (18%)	0.67
HFS grade 2	1 (11%)	2 (2%)	0.19
Hematologic toxicity grade 2	1 (11%)	9 (7%)	0.51
Median cycle of first grade 3 toxicity (IQR)	1.5	1.5 (1–2)	
Fluoropyrimidine-related hospitalizations	1 (11%)	6 (5%)	0.39

In the first three cycles, toxicity outcomes were compared between non-variant carriers and variant carriers who had treatment adjusted based on genotype (Table 4). All nine dose-reduced variant carriers and 127 (74%) of 178 non-variant carriers had completed three cycles of chemotherapy or completed their regimen before cycle 3 (Figure 2). Variant carriers given *DPYD* results received a median relative dose intensity of 50% (IQR 45%–74.5%). Most non-variant carriers received the standard dose, with a median relative dose intensity of 100 (IQR 92%–100%).

Of nine *DPYD* variant carriers receiving *DPYD*-guided fluoropyrimidine dose reductions, two experienced severe fluoropyrimidine-related toxicity compared to 14% in non-variant carriers receiving standard doses (Table 4). One (11%) variant carrier receiving a 50% dose reduction was hospitalized due to fluoropyrimidine-related toxicity, while six (5%) non-variant carriers were hospitalized.

### 3.1 Toxicity prevention with *DPYD*-guided dosing in variant carriers

Retrospectively discovered *DPYD* variant carriers experienced debilitating toxicities that led to immediate chemotherapy discontinuation after 1 cycle of the full dose. One received the full dose of capecitabine for cycle 1 for metastatic breast cancer. This led to severe oral mucositis, with severe pain limiting oral intake, and diarrhea, with an increase of more than seven stools per day (CTCAE grade 3). This patient also experienced HFS (CTCAE grade 2) and was hospitalized due to experiencing aortic thrombosis. Another received the full dose of capecitabine for cycle 1 for colorectal cancer, which led to prolonged diarrhea with an increase of over seven stools per day (CTCAE grade 3), and hospitalization.

Seven out of nine variant carriers tolerated chemotherapy well and were able to finish treatment with only Grade 1 toxicities. One variant carrier was scheduled to receive one cycle of capecitabine with concurrent radiotherapy for anal squamous cell carcinoma (stage IIIA). Even with a 50% dose reduction and normal renal function, this patient had to pause chemotherapy after 8 days due to severe thrombocytopenia (CTCAE grade 3) and was hospitalized. Platelet counts dropped to 35 × 10^9^/L from a baseline of 198 × 10^9^/L. After 1 week, this patient’s dose was further reduced to 25% of the full dose, and the patient completed cycle 1 without severe toxicity. Another variant carrier (c.557A>G) was a metastatic breast cancer patient who received the standard dose of capecitabine for cycle 1. The patient experienced diarrhea, with an increase of four stools a day and mucositis with ulceration and inflammation of the oral mucosa (CTCAE grade 2) and HFS (CTCAE grade 2) with peeling and blisters in the palms of the hands and soles of the feet. Despite a dose reduction in cycle 2, this patient’s HFS significantly worsened to grade 3, the most severe grade of HFS, leading to severe pain and ulceration. A further dose reduction was applied in cycle 3, leading to improved HFS (grade 2).

### 3.2 Dose tolerance of *DPYD* variant carriers

Two variant carriers received dose escalations, with one of the two resulting in severe toxicity. One was identified pre-emptively and the other was identified after discontinuing fluoropyrimidines due to toxicity. In the first patient, treated for metastatic pancreatic adenocarcinoma, results were returned during cycle 1, and a 50% dose reduction was applied for cycle 2, which the patient tolerated well. The dose was then increased to 67% of the full dose in cycle 3. This led to nausea that decreased oral intake (CTCAE grade 2), which was resolved with metoclopramide.

The other dose escalation was not successful. The patient was treated with capecitabine and oxaliplatin for esophageal adenocarcinoma (stage IV) before *DPYD* testing was available. In the first cycle, this patient was given 50% of the full dose due to impaired renal function. Because this patient had no toxicities at this dose for two cycles, the dose was increased to 75% of the full dose for cycle 3, which led to severe diarrhea, with an increase of seven stools per day (CTCAE grade 3) and HFS, with swelling and blistering of their palms that impeded their daily activity (CTCAE grade 2).

### 3.3 Implementation outcomes

Patients’ eligibility was confirmed with oncologists before their first oncology appointment. Out of 196 patients assessed, 12 did not end up on fluoropyrimidine-based regimens and were not enrolled (Figure 2). No patient declined testing.

*DPYD* testing was ordered for patients during their first appointment. The turnaround time for *DPYD* testing ranged from 2–10 days, with a median of 6 days. In most cases, patients were scheduled for chemotherapy 2–3 weeks after their first appointment. In 80% of patients, test results were available before patients started cycle 1. During the initial roll-out of testing, the patient care process was to start chemotherapy within 2–3 days of diagnosis. Given that tests were being run weekly to be most efficient with laboratory resources, a decision was made to order *DPYD* testing along with pre-chemotherapy bloodwork routinely done before the first appointment, such that *DPYD* results would routinely be available before fluoropyrimidine therapy was initiated. In the last month of our year-long study, only two patients out of 20 had their treatment delayed for up to a week to accommodate for the test turnaround time.

To facilitate the integration of *DPYD* results into dosing decisions, a dosing table was created to provide guidance for dose reductions corresponding to predicted activity scores as published in clinical guidelines (Table 2). Oncologists were notified that the dosing guidance could be found in the BC Cancer Drug Manual, an online resource maintained by the Provincial Health Services Authority. In addition to drug information, this resource contains patient handouts that were routinely referenced before patients were scheduled for chemotherapy.

## 4 Discussion

*DPYD* variant carriers are more vulnerable to adverse fluoropyrimidine reactions due to being exposed to elevated systemic levels of 5-fluorouracil (Amstutz et al., 2011). Although no pharmacokinetic analyses were done for this study, other pharmacokinetic studies from prospective *DPYD* testing trials have shown that dose reductions help establish therapeutic concentration levels, allowing variant carriers to be treated with fluoropyrimidines at an exposure comparable to non-variant carriers receiving standard doses (Deenen et al., 2016; Henricks et al., 2018). However, the authors noted that there is large interindividual variability in the exposure of capecitabine and its metabolites, so pharmacokinetic results should be interpreted cautiously (Meulendijks et al., 2015).

*DPYD*-guided dose reductions allowed seven of nine variant carriers to be more safely treated with fluoropyrimidines and therefore likely prevented dose-related toxicity. The incidence of severe toxicity in *DPYD* variant carriers treated at standard doses is over 70% (Meulendijks et al., 2015; Deenen et al., 2016). In this study, two out of nine *DPYD* variant carriers still experienced grade 3 toxicity. A *DPYD*-guided dose reduction was especially significant for the patient hospitalized after just 8 days of initiating capecitabine. Even at a 50% dose, the patient’s platelet count dropped 82% to 35 × 10^9^/L from a normal baseline. At a greater than 50% dose reduction, this patient was able to finish their course of adjuvant capecitabine with concurrent radiotherapy. For anal squamous cell cancer, pre-operative capecitabine and radiotherapy treatment were important to achieve a complete clinical response, which studies have shown to happen in 89.7% of patients (Meulendijks et al., 2014). This case influenced oncologists’ views on the necessity of *DPYD* testing and dose reductions for variant carriers. Most did not initially believe that pre-treatment *DPYD* testing was necessary, a sentiment echoed in a survey of US medical oncologists where only 32% of responders would recommend testing (Koo et al., 2022). However, due to the severity of this patient’s toxicity within the first week of treatment, oncologists accept that *DPYD* testing likely prevented lethal fluoropyrimidine toxicity. As a group, oncologists are now more willing to follow dose reduction guidelines for variant carriers. Critical to the use of guidelines were reminders that fluoropyrimidine patients who are variant carriers likely have higher toxic concentrations than those who are not variant carriers.

In non-variant carriers, 14% experienced severe fluoropyrimidine toxicity, which was in the expected range of 10%–30% (Mikhail et al., 2010; Froehlich et al., 2014; Meulendijks et al., 2016b; Barin-Le Guellec et al., 2020). The most common type of toxicity experienced was gastrointestinal toxicity (8%), then hematological toxicity (5%) (Table 4), frequencies also reflected in the literature (Meulendijks et al., 2016b). *DPYD* variants are known to be a major contributor to severe fluoropyrimidine toxicity in the first three cycles (Froehlich et al., 2014). *DPYD* variant carriers were not only more likely to experience adverse fluoropyrimidine events, but they also experienced toxicity in multiple categories, compounding the risk of therapy delay and cessation. This, taken into account, along with the fact that two out of nine variant carriers experienced grade 3 toxicity in cycles 1 and 2 despite reduced doses, reflects how variant carriers are substantially more at risk for fluoropyrimidine toxicity (Froehlich et al., 2014).

After *DPYD*-guided dosing, guidelines suggest subsequent titrating subsequent doses to toxicity (Amstutz et al., 2018). However, oncologists requested further guidance on dose escalations in variant carriers who tolerated reduced doses. One of the patients experienced nausea in cycle 3 after a dose escalation from 50% to 67%, which was resolved through anti-emetics. Another patient, whose *DPYD* status was unknown at the time of treatment, experienced severe diarrhea after their capecitabine dose was increased from 50% to 75% dose. If this patient’s variant carrier status had been known beforehand, a smaller dose increase might have been more successful. One study examined tolerance-based dose escalations of capecitabine, where 11 heterozygous *DPYD* variant carriers tolerated a mean dose escalation of 8.5% (Kleinjan et al., 2019). More experience and data need to be collected on tolerance-based fluoropyrimidine dose increases in *DPYD* variant carriers to inform physicians on how much they can escalate the dose. Given the severity of toxicities and the risk of not being able to resume chemotherapy treatment, the guidelines were modified to suggest a dose increase of 10% if a variant carrier tolerated a reduced dose without toxicity for two cycles.

The above notwithstanding, the authors acknowledge a lack of data on dose escalations for *DPYD* variant carriers in the literature. Prospective *DPYD*-guided dose reduction studies did not report on the size of dose increases (Deenen et al., 2016; Henricks et al., 2018). Henricks et al. described that 5 out of 11 dose escalations for *DPYD* variant carriers were not tolerated, leading to subsequent dose reductions and treatment discontinuation. Although the sizes of dose increases were not mentioned, this data emphasizes the need to be cautious with dose escalations. There are studies that implemented a dose escalation algorithm through TDM with 5-FU. Kaldate et al. specified a maximum 30% dose increase per cycle if AUC is less than 8, with a target AUC of 20–30 (Kaldate et al., 2012). TDM algorithms have also not been established for capecitabine, and systemic levels of capecitabine and its metabolites were shown to be poor in predicting toxicity in phase III studies, leading the authors to recommend against TDM for capecitabine (Gieschke et al., 2003).

A limitation of this study was the lack of assessment of other personalized medicine approaches to determine DPD activity such as TDM and phenotyping. Used in tandem with genotyping, phenotyping and TDM could capture more patients at risk of fluoropyrimidine toxicity. TDM has been shown to be useful in improving the pharmacokinetic interindividual variability and safety of 5-FU (Yang et al., 2020). TDM could also prevent underdosing in dose-reduced *DPYD* variant carriers and would be a way to escalate doses in those that tolerate reduced doses safely. Challenges to widespread TDM implementation include the instability of 5-FU in blood and the variability in measurements due to differences in the timing of blood samples (Hamzic et al., 2018; Hamzic et al., 2020a; Schneider et al., 2021).

There are also challenges with phenotyping. In a prospective study of DPD phenotyping with plasma uracil levels, pre-treatment levels varied between institutions and did not correlate with the DPD activity in peripheral blood mononuclear cells (de With et al., 2022). Phenotyping involving peripheral blood mononuclear cells is also costly, time-consuming, and difficult to upscale due to its complexity and specialized equipment (Chazal et al., 1996; Knikman et al., 2021). Comparatively, *DPYD* genotyping is a simple and cost-effective first step to pre-emptively identify at-risk patients (Meulendijks et al., 2016a; Meulendijks et al., 2016b; Hodroj et al., 2021; Knikman et al., 2021; Schneider et al., 2021). Access to DPD phenotyping and TDM would have utility in refining fluoropyrimidine dosing, especially for rare patients with a DPD activity score of zero. None were observed in this study, but for these patients, clinical guidelines state to avoid fluoropyrimidines entirely. However, in cases when this is not possible, phenotyping and TDM may be helpful in determining a patient’s residual DPD activity (or ability to otherwise clear 5-FU) in order to establish a starting dose (Lunenburg et al., 2020).

In the prospectively tested cohort, 10 (5%) of patients were found to be variant carriers, a frequency that was expected based on the prevalence of variants in the testing panel (Amstutz et al., 2018). Previous studies reported the limitation that the guideline recommended *DPYD* variants to test for were predominantly validated in European populations. Therefore, in this study’s variant selection, variants impacting DPD function observed in non-European populations were added. This was the first *DPYD* study to prospectively test for c.2279C>T, a variant found in 1% of those with South Asian ancestry (Offer et al., 2014). This study was also the second to prospectively evaluate c.557A>G, a variant found in 3% of those with African ancestry (Offer et al., 2013; Elraiyah et al., 2017). This variant was included in another *DPYD* implementation protocol in the US (Reizine et al., 2020) but prospective results from *DPYD* testing trials have not been reported on this variant yet (Meulendijks et al., 2015; Boisdron-Celle et al., 2017; Wigle et al., 2021). One patient tested positive for c.557A>G, affirming the clinical value of adding it to the testing panel.

In this study, the testing program development and implementation took place at the same time, providing data that informed improvements in the workflow. Initially, physicians were not checking for *DPYD* status determination before initiating fluoropyrimidine-based chemotherapy. This was primarily due to adding a new testing program to an existing workflow that did not include testing. Oncologists were accustomed to booking bloodwork and chemotherapy for some patients before their first appointment. While a turnaround time of two days would eliminate treatment delays, this is not the most efficient and cost-effective way to offer testing to a large population. To work around this limitation, physicians, more familiar now with the testing process, have begun ordering tests before a patient’s first appointment and in a few cases, adjusting chemotherapy start dates by a few days to accommodate the turnaround time.

A single physician may treat many patients without a single positive test result, which could lead to reduced confidence in the benefits of genotyping (Begre et al., 2022). However, people do have rare variants and, as a result, suffer from severe toxicities. In this cohort, there was a DPYD*13 variant carrier, a variant with a prevalence of 0.01% in those of European ancestry (Table 1).

Proactive engagement between the research team and oncologists led to high enrolment in the study, which prospectively screened all BC Cancer Vancouver site patients who were expected to start on a fluoropyrimidine-based regimen. Having a research team member on site was crucial for identifying patients suitable for the study and encouraging enrolment. Due to the high volume of clinic patients starting fluoropyrimidines, the research team member was needed to actively screen and consent patients. An on-site research assistant was crucial in the first 9 months to ensure test uptake and standardized outcome reporting. When introducing a new test, success is enhanced by the presence of dedicated personnel to help integrate it into existing workflows until it becomes routine for clinicians. However, this role is likely not necessary for the long-term maintenance of a pharmacogenetic testing program. After 9 months, we transitioned to integrating test requisition links into the electronic medical record to remind physicians to order *DPYD* testing. Testing continues at the same rate and penetrance as before.

A strength of this study is that it is a real-world study that provides experience in pharmacogenetic testing implementation. However, oncologists were not blinded to patient genotype when collecting toxicity outcomes, potentially leading to bias in toxicity staging. In addition, as genotyping for only six *DPYD* variants was implemented, not all DPD-deficient patients could be captured. Now that an in-house genotyping platform has been established, there is an opportunity to add, in the future, other variants that may impact fluoropyrimidine toxicity. Of interest would be the inclusion of a genetic variant in *TYMS* (rs45445694), which encodes 5-FU’s target thymidylate synthase (TS). This variant has been associated with increased risk for the common capecitabine-related toxicity hand-foot syndrome (OR 1.32, *p* < 0.0001) (Hamzic et al., 2020b). Other variants could also explain the severe toxicity seen in the variant carrier in this study who already received the recommended dose reduction. However, the effect sizes are small and expert guidelines do not recommend them currently. It may be that in time, stronger evidence is forthcoming to justify inclusion. In addition, pharmacogenetic variants relevant to other medications patients often take during chemotherapy may be of interest, such as *TPMT and NUDT15* testing to prevent thiopurine-induced myelosuppression, or *CYP2D6* testing to prevent metoclopramide-related adverse events in poor metabolizers (Relling et al., 2011; Livezey et al., 2014).

A potential limitation of this study was the lack of a control group, making it more difficult to observe the benefits of *DPYD* testing. However, it was considered unethical to have a control cohort, as a previous prospective clinical study that had a group without dose individualization was terminated due to a *DPYD* variant carrier in the group dying from treatment toxicity (Boisdron-Celle et al., 2017). Although this study’s sample size did not allow for a statistically relevant number of dose-reduced variant carriers to compare to patients receiving the full dose, other large prospective studies have shown that *DPYD*-guided dose reductions in variant carriers result in similar toxicity levels to non-variant carriers receiving standard doses (Henricks et al., 2018; Wigle et al., 2021). Taken together, these results have led to the widespread adoption of *DPYD* testing by oncologists province-wide.

This study shows how *DPYD* testing can be implemented, integrated into an existing clinical workflow, and can become the standard of care. In conclusion, taken together with the primary literature, the results support that *DPYD*-guided dosing is a feasible approach to reducing the risk of severe fluoropyrimidine toxicity in *DPYD* variant carriers.

## Data Availability

The raw data supporting the conclusion of this article will be made available by the authors, without undue reservation.
