# Supplementary material for: Implementation of pharmacogenetic testing in oncology: DPYD-guided dosing to prevent fluoropyrimidine toxicity in British Columbia
Source: Front Pharmacol. 2023 Sep 8;14:1257745. doi: 10.3389/fphar.2023.1257745 (PMC10515725; doi:10.3389/fphar.2023.1257745)
Supplement: Supplementary file 1 [file Table1.DOCX]

**Supplementary Appendix – EXAMPLE OF CLINICAL REPORTS**

**POSITIVE**

**REASON FOR REFERRAL:** DPYD mutation screen, query DPYD genotype

**SUMMARY:** Heterozygous for reduced function allele

**VARIANTS IDENTIFIED:**

**VARIANT 1:** DPYD:c.1236G>A, p.Glu412= [HapB3, reduced function allele]

**VARIANT 2:** None

**INTERPRETATION:**

This patient is heterozygous for a reduced function DPYD allele (see above). This result predicts for reduced DPD activity; suggesting a reduced initial dose of 5FU/capecitabine may be warranted.

This analysis does not detect all variation associated with 5FU toxicity. The presence of a second allele further reducing DPD function can, therefore, not be excluded. The present result should be considered accordingly.

Each of this patient's first degree relatives is minimally at 50% risk of also harbouring this change.

___________________________

Predicted CPIC activity Score: 1.5 (range: 0 - no activity -> 2.0 full activity)

Predicted CPIC DPD phenotype: intermediate metabolizer

The implications of this result on 5FU dosing for this patient are available through the BC Cancer – Drug Index - Capecitabine ordering.

**NEGATIVE**

# **REASON FOR REFERRAL:** DPYD mutation screen, query DPYD genotype

# **SUMMARY:** No variants identified

**VARIANTS IDENTIFIED:**

**VARIANT 1:** None

**VARIANT 2:** None

**INTERPRETATION:**

This analysis did not detect any variants which would suggest an a priori dose adjustment.

This analysis does not detect all variation associated with 5FU toxicity. The presence of an allele associated with reduced DPD function can, therefore, not be excluded. The present result should be considered accordingly.

___________________________

Predicted CPIC activity Score: 2.0 (range: 0 - no activity -> 2.0 full activity)

Predicted CPIC DPD phenotype: normal metabolizer

The implications of this result on 5FU dosing for this patient are available through the BC Cancer – Drug Index - Capecitabine ordering.
